# Prevalence and risk factors of preoperative anemia in patients undergoing elective orthopedic procedures in Northwest Ethiopia: a multicenter prospective observational cohort study

**DOI:** 10.1186/s13037-023-00373-w

**Published:** 2023-12-04

**Authors:** Getachew Mekete Diress, Gebremariam Ayele

**Affiliations:** Department of Anesthesia, School of Medicine, College of Health Sciences, Debre Tabor University, PO Box 272, Debre Tabor, Ethiopia

**Keywords:** Elective orthopedic surgery, Preoperative anemia, risk factors

## Abstract

**Background:**

Preoperative anemia is a common hematologic problem in major orthopedic surgery in developing countries. It is a condition in which the number and size of red blood cells are insufficient to meet the body’s physiologic needs, consequently impairing the capacity of the blood to transport oxygen to the body. Preoperative anemia is common in elective orthopedic surgical patients and is an independent risk factor for perioperative morbidity and mortality. This study aimed to assess preoperative anemia prevalence and risk factors in patients undergoing elective orthopedic procedures.

**Method:**

A multicenter prospective observational cohort study was conducted from June 01 to August 30, 2022. A systematic random sampling technique was used to select the study unit. Data were collected using a structured questionnaire. Descriptive statistics were expressed in percentages and presented with tables and figures. Binary logistic regression was used to see the association between independent and dependent variables. A P-value < 0.05 was considered statistically significant.

**Result:**

Preoperative anemia’s prevalence and risk factors in patients undergoing elective orthopedic procedures was 24.1[95%CI= (18.2–30.6)]. Multivariable logistic analyses showed that low monthly income level [AOR:5,95%CI:(1.36–7.98)], patient with cancer [AOR:3.4,95%CI:(3.7–8.84)], patient with malaria infectious [AOR: 3.2,95%CI:( 1.13–8.91)], patient with anti-retroviral therapy [AOR: 5.2,95%CI:( 1.8-11.04)], and previous history of surgery [AOR:1,95%CI(1.43–2.4)], were factors significantly associated with preoperative anemia.

**Conclusion:**

The prevalence of preoperative anemia among adult patients who underwent elective orthopedics procedures was high. Low Monthly income, patients with cancer, patient with malaria infection, and patients with anti-retroviral therapy, previous histories of surgery were found significantly associated with preoperative anemia. So, we recommend to health professional’s early identification, diagnosis and treatment of preoperative anemia should be done to reduce the risks of anemia and related adverse outcomes.

## Introduction

Anemia is a major health problem globally, which can cause a vicious impediment to the quality of life, mortality, morbidity, and socioeconomic progress of individuals especially in developing countries [1, 2]. Preoperative anemia is a common condition, affecting almost a third of the global population, and it is the most frequently observed impairment worldwide according to the Global Burden of Disease study [3].

According to the world health organization anemia can be defined as the reduction of a hemoglobin (Hb) level below 12 g/dL for non-pregnant women and 13.0 g/dL for men [4]. Anemia is associated with increased morbidity and mortality in women, child growth faltering, impairment of cognitive function, increased chances of various kinds of infection, and loss of productivity from impaired work capacity resulting in a substantial economic burden to the family and entire population [5, 6].

Pre-operative anemia is a powerful indicator of the need for blood transfusion; however, transfusion itself is independently associated with increased length of stay, surgical complications, and increased morbidity and mortality in orthopedic patients [7, 8].

Pre-operative anemia is independently associated with an increased risk of adverse outcomes following surgery; increased length of intensive care and prolonged hospital stays; perioperative complications; and increased mortality and morbidity of patients [9, 10].

Preoperative anemia (PA) is a risk factor for poor postoperative outcomes, increases the risk of oxygen depletion, and increased morbidity and mortality in orthopedic surgery [11]. Elective major orthopedic surgery (MOS), including knee and hip replacement and instrumented spinal fusion, is associated with substantial perioperative blood loss [12].

In orthopedics surgery, preoperative anemia is a common hematologic condition and an independent risk factor for perioperative morbidity and mortality. When compared to patients who are not anemic, anemia increases the chance of death from respiratory (respiratory failure and hypoxia), cardiac (myocardial infarction, congestive heart failure, and cardiac arrest), septic, multi-organ failure, and hemorrhagic [13].

According to different study the prevalence of preoperative anemia in orthopedics surgery range from 7 to 35% [14]. Pre-operative anemia prevalence varies by age, gender, comorbidities, surgical indication, lifestyle, and socioeconomic level, as well as the criteria used to define anemia [15, 16].

## Methods and materials

### Study area

The study was conducted at Debre Tabor comprehensive specialized Hospital and Felege Hiwot comprehensive specialized Hospital in the Amhara regional state of Northwest Ethiopia. All of these Hospitals have operation rooms that give services on elective and emergency bases. Approximately 15,000–20,000 patients undergo surgery in these hospitals per year. These referral hospitals were providing services for a population of around 30 million populations. These hospitals provide services like diagnosis, and treatment, for different procedures.

### Study design and period

A multicenter prospective observational cohort study was conducted from June 01 to August 30, 2022.

### Source population

All adult patients who were scheduled for major elective orthopedic surgery who underwent surgery in Amhara Regional State Governmental Hospitals of Ethiopia.

### Study population

Selected patients who underwent major elective orthopedic surgery in selected Amhara regional state governmental Hospitals of Ethiopia during study period.

### Inclusion and exclusion criteria

Study participants who were included in this study selected all patients 18 years and above who underwent major orthopaedics surgery were included. No preoperative haemoglobin record, Patient refusal, day-case surgery, and Patient on treatments of anaemia would be excluded from the study.

### Dependent variable

Preoperative anemia of major elective orthopedic surgery.

### Independent variables

Socio-economic and demographic variables (age, sex, BMI, ASA status, educational status, occupation, residence), comorbidity (peptic ulceration, myocardial infarction, hypertension, HIV, malaria, diabetes mellitus, malignancy, asthma, and renal disease), medications (chronic use of non-steroidal anti-inflammatory drugs (NSAID), antiretroviral therapy, and chemotherapy drugs.

### Sample size and sampling technique

At the national level, there is no documented information on the prevalence of preoperative anemia in orthopedics surgery and its associated factors. Using the finite population correction formula, the sample size was estimated by assuming a 0.5 prevalence of preoperative anemia in orthopedics surgery and a 5% margin of error at a 95% confidence interval using the following calculations.

$${\rm{n}}\,{\rm{ = }}\,{\left( {{{\rm{Z}}_{{\rm{\alpha /2}}}}} \right)^{\rm{2}}}{\rm{P}}\,\left( {{\rm{1 - P}}} \right){\rm{/}}{{\rm{d}}^{\rm{2}}}$$

Where; n = sample size Z = confidence interval (1.96) P = estimated prevalence (0.5) d = margin of sampling error to be tolerated (0.05) & ἀ= 5%.

$${\rm{n}}\,{\rm{ = }}\,{\left( {{\rm{1}}{\rm{.96}}} \right)^{\rm{2}}}\,{\rm{ \times }}\,{\rm{0}}{\rm{.5}}\,\left( {{\rm{1}} - \,{\rm{0}}{\rm{.5}}} \right){\rm{/}}\,{\left( {{\rm{0}}{\rm{.05}}} \right)^{\rm{2}}}\,{\rm{ = }}\,{\rm{384}}$$

The total number of adult elective orthopedics surgery performed in the hospital annually was below 10,000 and we found only average of 92 elective orthopedics surgery procedures done per a month by reviewing the operation registry and we were taken three consecutive months with similar to our data collection month. So, we were taken the three months totally we were taken 276. So we decided to apply reduction formula to obtain an achievable sample size.

nf = n/ (1 + n/N), N = 276…. correction formula for population less than 10,000.

So, nf = 384/ (1 + 384/276) = 160.6 Correction formula for population less than 10,000. Major elective orthopedic surgery patients.

We added 10% of nf for the non-response rate; (i.e., 160.6 + 16 = 176.6); As a result, a total of 177 adult major elective orthopedic surgery patients were included in this research.

Finally a systematic sampling technique was used to obtain the required sample size. The second case was chosen by lottery, and every k^th^ patient was chosen for the study period.

$${\rm{k}}\,{\rm{ = }}\,{\rm{N/n,}}\,{\rm{where}}\,{\rm{n}}\,{\rm{ = }}\,{\rm{total}}\,{\rm{sample}}\,{\rm{size}}\,{\rm{ = }}\,{\rm{177}}$$

N = population the last three months 276, sample interval = 276/177 = 1.56 ≈ 2.

Therefore, the sampling interval was two and the first study participant was selected using the lottery method from the daily surgery list of major elective orthopedics scheduled case.

### Data collection instruments and procedures

Data was collected by using an English version structure questionnaire taken from studies and translated to the Amharic language. The data collection procedure includes chart review and patient interview using a structured questionnaire. Data was collected from patients or caregivers by using a structured checklist questionnaire. Hemoglobin measurements were obtained from the patient’s medical records by reviewing charts. The data was collected by three trained BSc anesthetists after taking training on how to collect the questionnaire.

### Data quality assurance

Pretest was done to ensure the quality of data in 18 (10%) of the sample size) patients from other hospitals who were not included in the main study. Then, the necessary corrections were done accordingly to the questionnaire for the main study. Three days of training were given to the data collector and supervisor on the aim and objective of the study, the supervision, and the data collection process. The collected data were checked for completeness, accuracy, and clarity. Incomplete data were discarded and counted as non-response. Daily supervision and feedback were given by the principal investigator and supervisor during the data collection period.

### Data entry, analysis, and interpretation

The collected data were coded, entered into the Epi-data software (version 7) for cleaning errors, and analyzed by SPSS version 26. Descriptive statistical analyses were performed and presented with frequency, percentage, median, mean, and standard deviation. Hosmer and Lemeshow test was used to assess the goodness of fit. Variables with a p-value of less than < 0.2 in the Bivariable logistic analysis were fitted into a multivariable logistic regression analysis. The associations between the independent variables and dependent variables were determined at a 95% confidence interval with the chi-squared test, bivariate, and multivariate binary logistic regression, and presented in crude and adjusted odds ratio. In multivariable logistic regression analysis, variables with a p-value < 0.05 were considered statistically significant.

### Ethical consideration

Ethical clearance was received from the ethical reviewing committee and permission to conduct this research was obtained from the research and community service coordinator office of Debre Tabor University with the reference number CHS/1799/2014. Written informed consent was presented and obtained from each study participant according to the principles of the Helsinki Declaration. The Declaration of Helsinki was considered and principles and recommendations have been used.

### Operational definition

#### Anemia

is reduction of a hemoglobin (Hb) level below 12 g/dL (hematocrit < 36%) for nonpregnant women and 13.0 g/dL(hematocrit < 39%) for men [17].

#### Mild anemia

is hemoglobin (Hb) between 11 and 11.9 g/dL in non-pregnant women. **Moderate**: Hemoglobin 8.0-10.9 g/dL. **Severe**: Hemoglobin less than 8.0 g/dL [18].

#### Adult patient

- age of patient 18 years and above years for both genders.

#### Preoperative Hb

The most recent Hb assessed within 28 days before surgery was considered [19].

#### Orthopedic injury

is defined as an injury affecting the musculoskeletal system, which includes injuries to bones, joints, ligaments, tendons, muscles, and nerves.

#### Multiple sites fracture

defined as any types of fractures at two or more sites of the musculo-skeletal system.

#### Orthopedics surgery

- is a type of surgery in which a medical professional, such as an orthopedist or orthopedic surgeon, performs surgery on the bones, joints, and ligaments of the human body to correct disorders [20].

## Result

### Socio-demographic characteristics of study participants

A sample of 170 study participants was involved in this study with a response rate of 96.5%. The majority of participants in this study were males 110 (64.7%). Regarding age distribution, the majority of them were under the age group of 18–49 which account for 113 (66.5%). Of the individuals (65.9%) were rural and 102(60%) are literate. only some individuals (4.1%) were business owners (Table 1).

**Table 1:** Socio-demographic characteristics of study participants among major elective orthopedic surgery at North West Ethiopia, (n = 170)

Variables	Categories	Frequency	Percent (%)
Sex	Male	110	64.7
	Female	60	35.3
BMI	< 18.5	10	5.9
	18.5–24.9	152	89.4
	> 25	8	4.7
Residence	Urban	58	34.1
	Rural	112	65.9
Educational level	Literate	102	60
	Illiterate	68	40
Monthly income	High	7	4.1
	Medium	23	13.5
	Low	140	82.4

### Clinical characteristics of study participants

Among the total collected 145(85.29%) were ASA1, 12(12.06%,) were hypertension of these 5(41.67%), 1(0.59%) were malignancy, 10(5.88%) of these (50%) were anemic, 4(2.35%) of these 100(58.2%) had history of trauma and 68(40%) had history of previous surgery (Table 2).

**Table 2:** frequency of preoperative clinical characteristics of the patient among elective major orthopedics patients at North West Ethiopia, (n = 170)

Variables	Categories	Total(n)	Percent (%)
ASA	ASA1	145	85.29
	ASA2	23	13.23
	ASA3	2	1.18
Hypertension	Yes	12	7.06
	No	158	92.94
Malignancy	Yes	49	28.8
	No	121	71.2
DM	Yes	10	5.88
	No	160	94.12
Malaria	Yes	43	25.3
	No	127	74.7
Peptic ulcer	Yes	6	3.53
	No	164	96.47
Smoking	Yes	2	1.18
	No	168	98.82
Asthma	Yes	6	3.53
	No	164	96.47
HIV	Yes	2	1.18
	No	168	98.82
Renal failure	Yes	15	8.82
	No	155	91.28
Chemotherapy	Yes	20	11.76
	No	150	88.24
NSAID	Yes	48	28.24
	No	122	71.76
ART medication	Yes	2	1.18
	No	168	98.82
History of Previous trauma	Yes	100	58.82
	No	70	41.18
History of Previous surgery	Yes	68	40
	No	102	60

### Magnitude of preoperative anemia

**Fig. 1:**
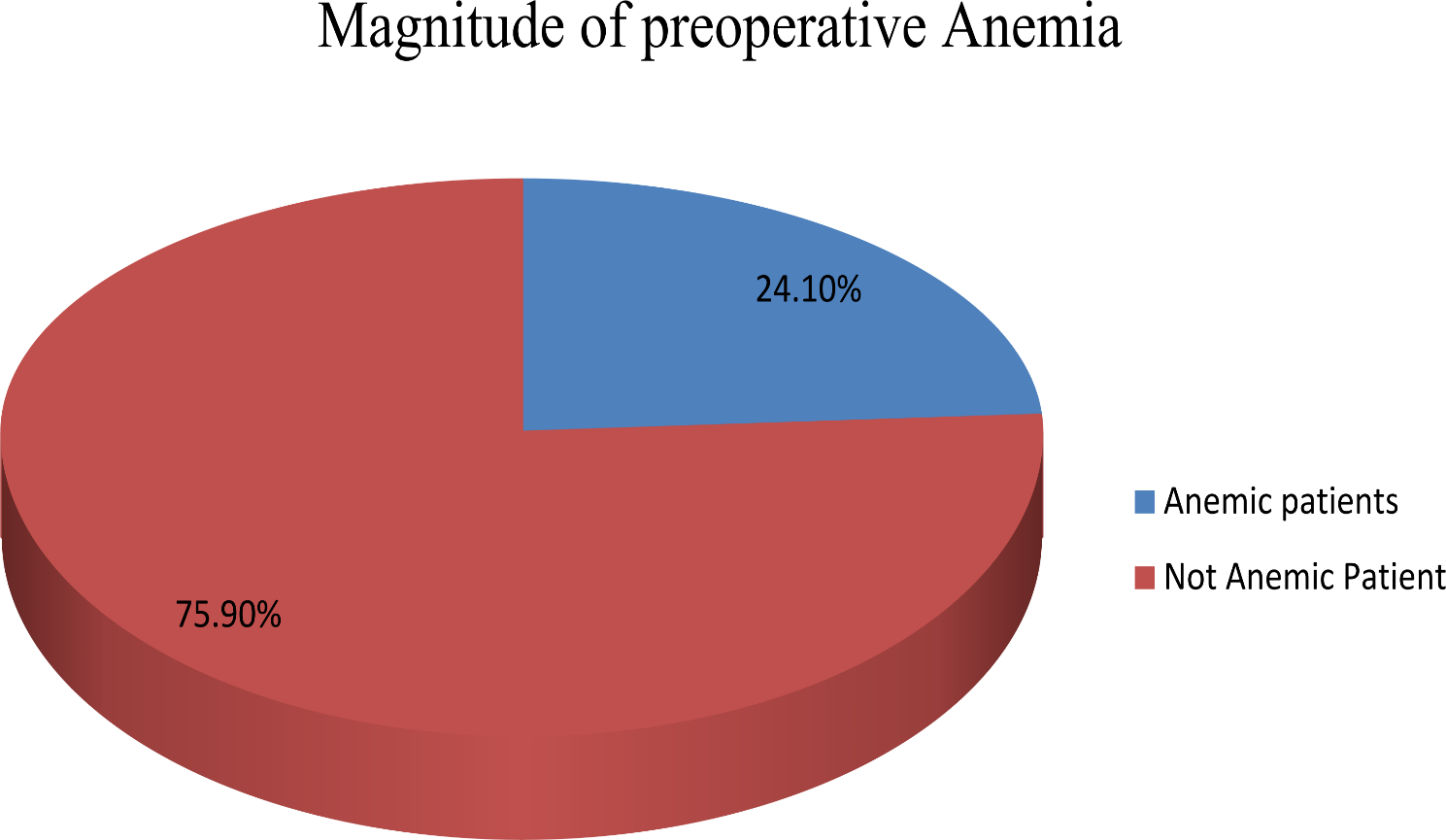
Magnitude of preoperative anemia among adult elective major orthopedics surgical patients

According to the study, the prevalence of anemia among elective major orthopedics patients was 41(24.1%) [95%CI= (18.2–30.6)].Regarding the severity of anemia, majority of 25(60.97%) elective orthopedic patients for anemia was mild anemia, 10(24.4%) was moderate anemia and 6 (14.63%) patient with severe anemia (Fig. 1).

### Factors associated with anemia

Multivariable logistic regression analysis shows that low monthly income, patients with cancer, patients with malaria infectious, patients with anti-retroviral therapy and a history of surgery were factors associated with preoperative anemia.

Patients who had low monthly income were 5 times [AOR: 5, 95%CI :( 1.36–7.98)] more likely to develop preoperative anemia than patients who were medium and high monthly income. Patients with cancer were 3.4 times [AOR: 3.4, 95%CI :( 3.7–8.84)] more likely to develop preoperative anemia than patients who had no cancer.

Patients with malaria infectious were 3.2 times [AOR: 3.2, 95%CI :( 1.13–8.91)] more likely to develop preoperative anemia than patients who had not malaria infectious. Patients with anti-retroviral therapy were5.2 times [AOR: 5.2, 95%CI :( 1.8-11.04)] more likely to develop preoperative anemia than patients who were not anti-retroviral therapy.

Pervious history of surgery were 1 times [AOR: 1, 95%CI (1.43–2.4)] more likely to develop preoperative anemia than patients who had not pervious history of surgery (Table 3).

**Table 3:** Bi-variable and multivariable binary logistic regression: Factors associated with preoperative anemia among major elective orthopedics surgical patients at North West Ethiopia, 2021, (N = 170)

Variable	Category	Anemia		COR(95%CI)	AOR(95%CI)	P value
		Yes	No			
Sex	Male	34(30.91%)	76(69.09%)	1	1	-
	Female	6(10%)	54(90%)	2.7(1.14–6.2 )	2.5(0.96–6.4)	0.061
Monthly income	High	1(14.3%)	6(85.7%)	1	1	-
	Medium	5(21.7%)	18(78.3%)	2.7(0.59–12.7)	3.6(0.49–6.9)	0.203
	Low	35(25%)	105(75%)	3.8(1.3–10.9)	5(1.36–7.98)	**0.015**
Patient with cancer	YES	25 (51%)	24(49%)	1	1	-
	NO	16(13.2%)	105(86.8%)	2.1(2.56–6.7)	3.4(3.7–8.84)	**0.002**
Chemotherapy	YES	31(30.39%)	71(69.61%)	1	1	-
	NO	9(13.24%)	59(86.76%)	2.5(0.75–8.31)	5(0.9–28.4)	0.060
Patient with malaria infection	Yes	28(65.12%)	15(34.88%)	1	1	-
	No	13(10.24%)	114(89.76%)	2.8(1.26–6.48)	3.2(1.13–8.9)	**0.027**
DM	Yes	5(50%)	5(50%)	2.4(0.46–12.5)	2.9(0.4–21.4)	0.275
	No	35(21.87%)	125(78.13%)	1	1	-
anti-retroviral therapy	Yes	34(34%)	66(66%)	4.4(1.8–10.7)	5.2(1.8–11.1)	**0.002**
	No	6(8.57%)	64(91.43%)	1	1	-
Pervious history of surgery	Yes	20(29.41%)	48(70.59%)	1.7(1.8–3.5)	1(1.43–2.4)	**0.001**
	No	20(19.61%)	82(80.39%)	1	1	-

**: significant (p-value < 0.05) **AOR**: adjusted odds ratio, **COR**: crude odds ratio **CI**: confidence interval, **I**: Reference.

## Discussion

This study reviled that the magnitude of preoperative anemia was 41 (24.1%) indicating a higher magnitude. Out of these, 25(60.97%) were develop mild anemia, 10(24.4%) were moderate anemia, and 6 (14.63%) patients developed severe anemia. The risk factors analyzed by multivariable logistic regression, low monthly income, patients with cancer and malaria infectious, patients with anti-retroviral therapy, and previous surgery history were significantly associated with preoperative anemia.

In this study, the magnitude of preoperative anemia was found to be lower when compared to the study that was conducted in the United States patients undergoing elective hips or knee arthroplasty showed that up to 35% of these patients were anemic before surgery [21].

But this finding is higher than a study conducted in previous research in hospital-based observation study of elective orthopedics procedures conducted in Seventeen centers in six European countries (14.1%) [22] major elective orthopedic surgery such as knee or hip arthroplasty or back surgery at University of Lausanne, in Switzerland 20.5% [23] elective orthopedic surgery in Sweden (21.5%) [24],elective orthopedic surgery in Australia (14.6%) [25].Possible cause for these variations could be due to differences in the cutoff value used to measure anemia and in sample size, date of hemoglobin, in clinical setups, sample size, and the sampling techniques used difference, when we used systematic random sampling, but they were used convenience sampling techniques.

In this study patient with low monthly income was significantly associated with preoperative anemia. This result was consistent with a study conducted by WHO that reported that 97% anemic population lives in low and middle-income countries [26–28]. The possible reason could be due to poor intake of iron or other nutritional deficiencies this result would affect hemoglobin synthesis and red blood cell production.

Patients who had malignancy or patients who were with cancer were significantly associated with preoperative anemia. This result was supported by other studies [29–32]. This possible reason could be due to directly or indirectly exacerbating anemia either by suppressing hematopoiesis through bone marrow infiltration or production of cytokines that lead to iron sequestration, inhibit release and synthesis of endogenous erythropoietin, reduce the response of erythroid progenitor cells to erythropoietin, which ultimately impair erythropoiesis.

In this study patients who had a history of malaria infection were significantly associated with preoperative anemia. This result was supported by other studies [33, 34]. The possible reason could be due to Malaria infection causing hemolysis of infected and uninfected erythrocytes and bone marrow dyserythropoietic which compromises rapid recovery from anemia.

Patients who were taken anti-retroviral therapy were significantly associated with preoperative anemia. This result was supported by a study conducted by Zerihun KW et al. [35–38]. The possible reason could be due to HAART medications would result, decrease production of endogenous erythropoietin, and hemolysis due to RBC autoantibodies.

Patients who had a history of previous surgery were significantly associated with preoperative anemia. This result was consistent with a study conducted by Y.E. Sim, and H.E. Wee et al. in Singapore and Switzerland [22, 39]. The possible reason could be post-operative reduced erythropoiesis and surgery-associated inflammation.

## Conclusion and recommendations

The magnitude of Preoperative anemia was high in this study. Therefore, patients undergoing elective orthopedic surgeries should be assessed early enough to allow for proper investigation and treatment before the scheduled procedure.

### Strength and limitation of the study

The strength of our study was multicenter data collection, so this was used for the generalization of the result and It would be helpful as a baseline for future researchers. The limitations of this study were, the sample size was slightly lower than the previous study and unable to get enough articles with similar study designs done in Ethiopia.

## Data Availability

The data of this study will be available from the corresponding author upon reasonable request. Search manuscript. Also, all authors read and approved the revised manuscript for publication.

## Acronyms and abbreviations

ART: Antiretroviral therapy
ASA: American society of Anesthesiologists
BMI: Body Mass Index
DCSH: Debre Tabor comprehensive specialized Hospital
DTU: Debretabor University
ETB: Ethiopian Birr
HAART: Highly Active Anti-retroviral therapy
Hb: Hemoglobin
HCP: Health Care Profession
HIV: Human immunodeficiency virus
IV: Intra Venous
MCH: Mean corpuscular hemoglobin
MCV: Mean corpuscular volume
NSAID: Non-steroidal anti-inflammatory drugs
RBC: Red blood cells
VTE: Venous thromboembolism
WBC: White blood cell
WHO: World Health Organization

## Declarations

### Provenance and peer review

Not commissioned externally peer-reviewed.

## References

[1] Rammohan A, Awofeso N, Robitaille M-C. Addressing female iron-deficiency anaemia in india: is vegetarianism the major obstacle? International Scholarly Research Notices, 2012. 2012.

[2] Muñoz M (2017). Pre-operative haemoglobin levels and iron status in a large multicentre cohort of patients undergoing major elective surgery. Anaesthesia.

[3] Goodnough LT (2011). Detection, evaluation, and management of preoperative anaemia in the elective orthopaedic surgical patient: NATA guidelines. Br J Anaesth.

[4] Ferraris VA (2012). Surgical outcomes and transfusion of minimal amounts of blood in the operating room. Arch Surg.

[5] Hong FS (2017). Prevalence and causes of preoperative anaemia in elective major surgery patients. Intern Med J.

[6] Marsicano D (2018). Preoperative anaemia and clinical outcomes in the South African Surgical Outcomes Study. South Afr Med J.

[7] Spahn DR (2010). Anemia and patient blood management in hip and knee surgery: a systematic review of the literature. J Am Soc Anesthesiologists.

[8] Jans Ø (2014). Role of preoperative anemia for risk of transfusion and postoperative morbidity in fast-track hip and knee arthroplasty. Transfusion.

[9] Musallam KM (2011). Preoperative anaemia and postoperative outcomes in non-cardiac surgery: a retrospective cohort study. The Lancet.

[10] Kotze A, Carter L, Scally AJ (2012). Effect of a patient blood management programme on preoperative anaemia, transfusion rate, and outcome after primary hip or knee arthroplasty: a quality improvement cycle. Br J Anaesth.

[11] Abdullah HR (2017). Association between preoperative anaemia with length of hospital stay among patients undergoing primary total knee arthroplasty in Singapore: a single-centre retrospective study. BMJ open.

[12] Rosencher N (2003). Orthopedic surgery transfusion hemoglobin european overview (OSTHEO) study: blood management in elective knee and hip arthroplasty in Europe. Transfusion.

[13] Muñoz M (2015). Pre-operative anaemia: prevalence, consequences and approaches to management. Blood Transfus.

[14] Dunne JR (2002). Perioperative anemia: an independent risk factor for infection, mortality, and resource utilization in surgery. J Surg Res.

[15] Muñoz M (2015). Fit to fly’: overcoming barriers to preoperative haemoglobin optimization in surgical patients. Br J Anaesth.

[16] Burton BN (2018). Optimizing preoperative anemia to improve patient outcomes. Anesthesiol Clin.

[17] Organization WH. Haemoglobin concentrations for the diagnosis of anaemia and assessment of severity. World Health Organization; 2011.

[18] Akbarpour E (2022). Anemia prevalence, severity, types, and correlates among adult women and men in a multiethnic iranian population: the Khuzestan Comprehensive Health Study (KCHS). BMC Public Health.

[19] Natera L (2015). Blood transfusion requirements in lower limb arthroplasties might be dramatically reduced if orthopaedic surgeons were concerned about preoperative anaemia. Eur Orthop Traumatol.

[20] Viola J (2015). Preoperative anemia increases postoperative complications and mortality following total joint arthroplasty. J Arthroplast.

[21] Heller LB, Shander A (2020). Preoperative anemia management: value-based care for orthopedic surgery. Techniques in Orthopaedics.

[22] Lasocki S (2015). PREPARE: the prevalence of perioperative anaemia and need for patient blood management in elective orthopaedic surgery: a multicentre, observational study. Eur J Anaesthesiology| EJA.

[23] Theusinger OM (2007). Treatment of iron deficiency anemia in orthopedic surgery with intravenous iron: efficacy and limits: a prospective study. J Am Soc Anesthesiologists.

[24] Wan S (2020). Clinical and budget impact of treating preoperative anemia in major orthopedic surgery—a retrospective observational study. J Arthroplast.

[25] Delaforce A et al. Preoperative anemia screening and treatment practices in patients having total joint replacement surgery: a retrospective, observational audit. J Blood Med, 2020: p. 259–65. 10.2147/JBM.S254116 PMC7418168 32821186

[26] Horton S, Ross J (2003). The economics of iron deficiency. Food Policy.

[27] Horton S, Ross J (2007). Corrigendum to:“ the Economics of iron deficiency“[Food Policy 28 (2003) 51–75]. Food Policy.

[28] Organization WH. Global health risks: mortality and burden of disease attributable to selected major risks. World Health Organization; 2009.

[29] Gaspar BL, Sharma P, Das R (2015). Anemia in malignancies: pathogenetic and diagnostic considerations. Hematology.

[30] Gandhi SJ (2017). Prevalence, comorbidity and investigation of anemia in the primary care office. J Clin Med Res.

[31] Lee EH, Otoukesh S, Nagaraj G (2019). Hemolytic anemia of malignancy: a case study involving signet ring cell metastatic breast cancer with severe microangiopathic hemolytic anemia. Case Rep Oncol.

[32] Kifle E et al. Prevalence of anemia and associated factors among newly diagnosed patients with solid malignancy at tikur anbessa specialized hospital, radiotherapy center, addis ababa, Ethiopia. Advances in hematology, 2019. 2019. 10.1155/2019/8279789 PMC6855075 31781226

[33] Amponsah G, Charwudzi A. Preoperative anaemia and associated postoperative outcomes in noncardiac surgery patients in central region of Ghana. Anesthesiology research and practice, 2017. 2017. 10.1155/2017/7410960 PMC5742513 29375620

[34] Organization WH. Global diffusion of eHealth: making universal health coverage achievable: report of the third global survey on eHealth. World Health Organization; 2017.

[35] Zerihun KW, Bikis GA, Muhammad EA (2019). Prevalence and associated factors of anemia among adult human immune deficiency virus positive patients on anti-retroviral therapy at Debre tabor Hospital, Northwest Ethiopia. BMC Res Notes.

[36] Volberding PA (2004). Anemia in HIV infection: clinical impact and evidence-based management strategies. Clin Infect Dis.

[37] Fekene TE (2018). Prevalence of cytopenias in both HAART and HAART naïve HIV infected adult patients in Ethiopia: a cross sectional study. BMC Hematol.

[38] Berhane Y, Haile D, Tolessa T. Anemia in HIV/AIDS patients on antiretroviral treatment at Ayder specialized hospital, Mekele, Ethiopia: a case-control study. J Blood Med, 2020: p. 379–87. 10.2147/JBM.S275467 PMC7585826 33117024

[39] Sim YE (2017). Prevalence of preoperative anemia, abnormal mean corpuscular volume and red cell distribution width among surgical patients in Singapore, and their influence on one year mortality. PLoS ONE.

